# Adenosine Receptors in Neuroinflammation and Neurodegeneration

**DOI:** 10.3390/cells14201585

**Published:** 2025-10-11

**Authors:** Veronica Salmaso, Silvia Menin, Stefano Moro, Giampiero Spalluto, Stephanie Federico

**Affiliations:** 1 Molecular Modeling Section (MMS), Department of Pharmaceutical and Pharmacological Sciences, University of Padova, Via Marzolo 5, 35131 Padova, Italy; silvia.menin.1@phd.unipd.it (S.M.); stefano.moro@unipd.it (S.M.); 2 Department of Chemical and Pharmaceutical Sciences, University of Trieste, Via Licio Giorgieri 1, 34127 Trieste, Italy; spalluto@units.it

**Keywords:** adenosine, adenosine receptors, caffeine, GPCR, purinergic system, neuroinflammation, neurodegenerative diseases, Alzheimer’s disease, Parkinson’s disease, Huntington’s disease, Amyotrophic lateral sclerosis

## Abstract

Adenosine plays a crucial role in various pathophysiological conditions, including neuroinflammation and neurodegeneration. Neuroinflammation can be either beneficial or detrimental to the central nervous system, depending on the intensity and duration of the inflammatory response. Across a wide range of brain disorders, neuroinflammation contributes to both the onset and progression of disease. Notably, neuroinflammation is not limited to conditions primarily classified as neuroinflammatory but is also a key factor in other neurological disorders, including life-threatening neurodegenerative diseases. All four adenosine receptor subtypes (A_1_, A_2A_, A_2B_, and A_3_) are implicated, to varying degrees, in these conditions. This review aims to summarize the roles of individual adenosine receptor subtypes in neuroinflammation and neurodegenerative diseases, emphasizing their therapeutic potential. While some therapeutic applications are well-established with clinically approved drugs, others warrant further investigation due to their promising potential.

## 1. Introduction

### 1.1. Adenosine and Adenosine Receptors

Adenosine is an endogenous purine nucleoside. Beyond its role in energy metabolism (it is the final product of the catabolism of the energetic molecule by definition, adenosine triphosphate (ATP)), adenosine modulates numerous processes in the human organism through the recognition of its receptors (i.e., A_1_, A_2A_, A_2B_, and A_3_) and consequent cell signaling and behaves as a neuromodulator in the central nervous system (CNS). It exerts a cytoprotective effect in response to stress, thus, for instance, limiting the damage provoked by excessive inflammatory response; for this reason, it has been defined as a retaliatory metabolite [1].

In physiological conditions, the extracellular concentration of adenosine falls in the 20–300 nM range, rising to the order of 10 µM in case of metabolic stress and hypoxia, which are well-known to trigger inflammation [2,3]. This effect has been particularly studied in the CNS, where hypoxia can lead to a 30 µM extracellular adenosine concentration [3,4,5]. The extracellular adenosine is produced by the enzymatic catabolism of adenosine nucleotides by means of different ectonucleotidases [6], and it is also generated intracellularly and exported through nucleoside transporters or as a result of cell damage [7,8,9].

**Figure 1:**
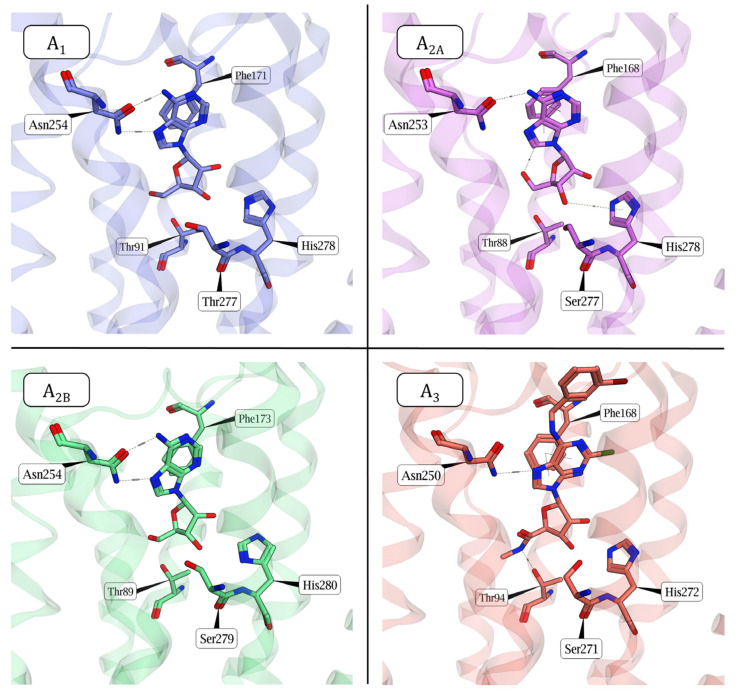
Experimental structures (cryo-electron microscopy) of the four active-state adenosine receptor (AR) subtypes bound to an agonist. A_1_ (PDB ID: 7LD4, blue), A_2A_ (PDB ID: 9EE8, magenta), and A_2B_ (PDB ID: 8HDP, green) ARs are bound to the endogenous agonist adenosine, while A_3_ AR (PDB ID: 8X17, orange) is bound to Namodenoson (no adenosine-bound structure available to date). The figure shows a focus on the orthosteric binding pocket of the receptors, and it shows the conservation of key amino acids involved in agonist binding.

Adenosine exerts its effects through its four adenosine receptors (ARs) that are class A G protein-coupled receptors (GPCRs), structurally characterized by the typical seven-helices transmembrane bundle connected by three intracellular and three extracellular loops of various lengths, an N-terminal region facing the extracellular milieu and an intracellular C-terminal region, constituted by an amphipathic helix (helix 8) and a disordered tail. Currently, at least one experimental tridimensional structure has been released for each receptor subtype (some examples are reported in Figure 1), with A_2A_ AR being the first and most explored one.

The four AR subtypes present high sequence identity, especially in the transmembrane helices (TM) region, with the couple A_2A_-A_2B_ characterized by the highest transmembrane sequence identity (66%), followed by A_1_-A_3_ (57%), A_1_-A_2A_ (55%), A_1_-A_2B_ (52%), A_2A_-A_3_ (47%), and A_2B_-A_3_ (45%) (values computed through https://gpcrdb.org/ (accessed on 22 August 2025) [10], focusing on TM1-7). The similarity among the receptors is especially high in the orthosteric binding pocket, where Asn6.55, Phe45.52^EL2^, Thr3.36, Ser7.42 (replaced by Thr in A_1_), and His7.43 (numbered according to Ballesteros–Weinstein [11]) are key residues for agonists binding (Figure 1). It is worth noting that few residues discriminate the orthosteric binding pocket of ARs. For instance, A_3_ presents the following mutations compared to the other three AR subtypes: Val3.32Leu, Gln3.37His, Asn5.42Ser, Val5.47Ile, His6.52Ser, and Thr6.58Ile. Also, the following residues show some subtype variability: Val/Ala/Ala/Val 2.57 (respectively, in A_1_/A_2A_/A_2B_/A_3_ ARs), Asn/Ser/Ser/Ser 2.65, Leu/Leu/Val/Leu 6.51, Thr/Met/Met/Leu 7.35, and residues Glu170/Leu167/Leu172/Gln167 and Glu172/Glu169/Glu174/Val169, respectively before and after the conserved Phe45.52 on EL2. Additionally, major differences among AR subtypes affect the extracellular and intracellular regions of the receptors. A_2A_ presents an unusually long C-terminal tail, more than 80 residues longer than the other subtypes. Extracellular loop 2 (EL2), which has been observed to play a role in ligand recognition [12], is peculiarly different among the receptor subtypes: A_3_ is characterized by the shortest loop (24 residues) and A_2B_ by the longest one (34 residues), with A_1_ and A_2A_ being in the middle. All EL2s are connected to TM3 through a disulfide bond between the conserved Cys45.50^EL2^ and Cys3.25 on TM3, that is typical of class A GPCRs; moreover, two additional disulfide bonds connect EL2 to EL1 in the case of A_2A_ AR, causing some degree of rigidity to the loop, while the role of three additional cysteines in A_2B_’s EL2 still needs to be fully elucidated [13,14]. The A_1_ loop contains a long 3-turn α-helix positioned almost perpendicularly to the membrane leaflet; in A_2A_, EL2 forms a 2.5-turn α-helix parallel to the membrane. In the case of the A_3_ AR, EL2 contains a short 1.5-turn alpha helix, while the conformation of the long A_2B_ AR EL2 has not been solved yet. Also, EL3 is longest in the case of A_2B_, where it outscores by four, two, and one residues, respectively, A_3_, A_1_, A_2A_; a disulfide bond between EL3 and the tip of TM6 (Cys6.61) characterizes A_1_ and A_2A_.

**Figure 2:**
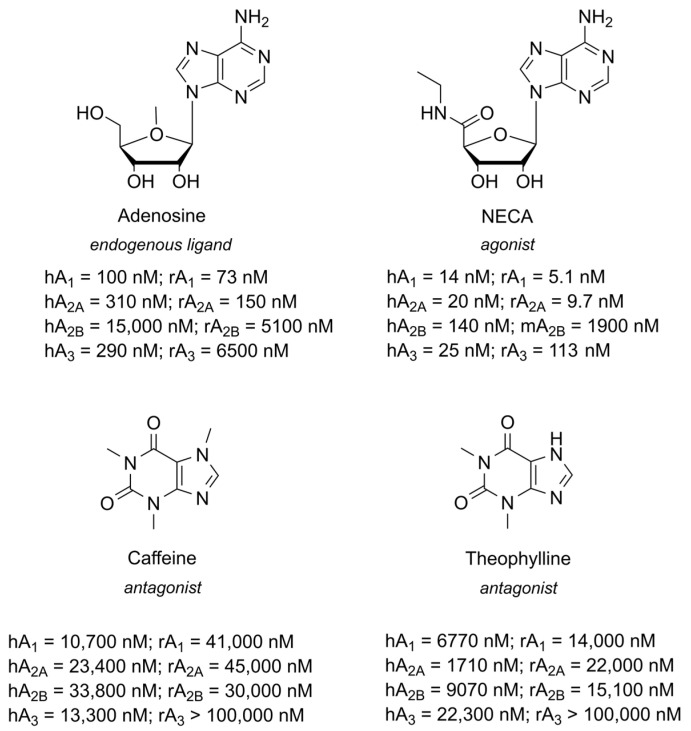
Structures of the endogenous ligand adenosine and of the non-selective agonist NECA and antagonists caffeine and theophylline.

Adenosine has a different affinity towards the four ARs. As reported in Figure 2, adenosine shows an affinity in the high nanomolar range towards A_1_, A_2A_, and A_3_ ARs. Instead, A_2B_ AR presents a considerable weaker affinity (µM) as compared to the other subtypes (medium to high nM) [15]. This behavior is observed both in humans and rats, except for the A_3_ AR subtype, for which adenosine displays a micromolar affinity at the rat compared to human receptor. In fact, a high interspecies difference is present between rA_3_ AR and hA_3_ AR [16].

Adenosine receptors are characterized by different tissue and cell distribution. A_1_AR is highly expressed in the CNS, especially in the brain cortex, cerebellum, and hippocampus. It is also highly distributed in the heart atria, eye, adrenal glands, adipose tissue, testis, kidney, liver, and others. A_2A_ AR has the highest expression levels in the spleen, thymus, leukocytes (lymphocytes and granulocytes), blood platelets, in various brain regions such as striatum, nucleus accumbens, and olfactory tubercles, olfactory bulb, heart, blood vessels, and lungs; A_2B_ AR in the cecum, colon, bladder, and blood vessels [17,18,19]. A_3_ AR is expressed in diverse tissues at relatively low levels, such as the testis, spinal cord, various brain regions, bladder, lung, adipose tissue, and whole blood [20]. Moreover, the four AR subtypes are also expressed in various immune cells [21] and overexpressed in cancer cells [22,23,24].

Receptor distribution is important because both basal and induced adenosine influence cellular behavior depending on the specific expression patterns of adenosine receptor subtypes within each cell type but also depending on the entire dynamic (temporal) process involving the receptor expression and the adenosine release [17,25,26]. Adenosine receptors couple to different G proteins and downstream intracellular signaling pathways. G proteins are the primary intracellular partner of the receptors, but they are also involved in other signaling cascades. For instance, all AR subtypes can engage mitogen-activated protein kinase (MAPK) pathways, but the mechanisms and outcomes of MAPK activation vary depending on the receptor subtype and cell context. Here, G proteins and further relevant AR interacting partners are reported, together with the organ distribution of the receptors [17,27].

A_1_ and A_3_ ARs couple to Gi proteins, whose activated Gαi subunits inhibit adenylyl cyclase and decreases intracellular cAMP levels with a negative effect on the downstream effectors. The Gβγ subunit activates phospholipase C (PLC), with a consequent increase in inositol 1,4,5-trisphosphate (IP3) and intracellular Ca^2+^ levels, which stimulate protein kinase C (PKC) and other calcium-binding proteins [17,27,28].

A_2A_ and A_2B_ ARs couple to Gs proteins, which activate adenylyl cyclase, increasing cAMP levels and in turn activating protein kinase A (PKA) and its downstream signaling pathways (such as cAMP response element-binding protein, CREB). The A_2A_ AR couples mainly to Gs in peripheral systems, but in the striatum, where the receptor is more abundant, it is coupled to Golf, which has a Gs-like behavior [17,27,28].

Pharmacological and signaling studies suggest that the A_2A_ receptor is the major subtype accounting for adenosine-induced mast cell tPA (tissue plasminogen activator) activity and provide valuable insights into the regulation of A_2A_ AR expression in macrophages in response to inflammatory stimuli [29,30].

A_2B_ and, at a high agonist concentration, A_3_ ARs can also couple to Gq proteins, which activates PLC [27].

In cardiac muscles and neurons, A_1_ AR can activate pertussis toxin-sensitive and K_ATP_ potassium ion channels and inactivate Q-, P-, and N-type calcium channels, influencing excitability [28].

ARs can also activate MAPKs, such as ERK1/2 (extracellular-regulated kinases, ERK) or p38 and JNK (c-Jun N-terminal kinase), thus promoting cell differentiation, survival, proliferation, and death. A_1_ can activate ERK1/2 through the Gi/oβγ subunit, A_2A_ can activate or inhibit ERK1/2 in a Gs-dependent or -independent manner, A_3_ can activate ERK1/2, and A_2B_ can activate ERK1/2 and also p38 and JNK [17].

Given the many roles of adenosine and its receptors and their broad distribution, developing drugs against adenosine receptors is challenging [31]. Moreover, pursuing selectivity is not an easy task, but still, the availability of numerous pan-ligands provides useful pharmacological tools for these receptors: few examples of pan-agonists (adenosine and NECA, N-ethylcarboxamidoadenosine) and pan-antagonists (caffeine and theophylline) are reported in Figure 2.

Few drugs have been approved so far acting on ARs. The A_2A_ AR agonist Regadenoson has been approved for myocardial perfusion imaging (MPI) and the A_2A_ AR antagonist Istradefylline for Parkinson’s disease [32].

Adenosine, the endogenous ligand of Ars, is also a drug used to treat supraventricular tachycardia (SVT) by slowing down electrical signals at the atrioventricular (AV) node, helping to restore a normal heart rhythm [33]. Caffeine, instead, is present in different medicinal specialties as an analgesic adjuvant in combination with other analgesics [34]. Also, theophylline, a non-selective AR antagonist, is a currently used drug in chronic obstructive pulmonary disease (COPD), but the therapeutic effect is mediated by the inhibition of phosphodiesterases [35].

### 1.2. Neuroinflammation

Neuroinflammation is a complex inflammatory reaction within the CNS. As any inflammatory reaction, it is characterized by the release of mediators of inflammation that, in the case of neuroinflammation, are produced by different cell types such as resident CNS glial cells (i.e., microglia and astrocytes), endothelial, and peripheral immune cells infiltrated into CNS. Other hallmarks of neuroinflammation are the increased permeability of the blood–brain barrier (BBB) and edema [36].

Neuroinflammation is a response to several insults, like infection, stroke, brain and spinal cord injury, and traumatic and chronic stressors. It has a beneficial effect as an initial inflammatory response to insults, acting as a protective mechanism to limit tissue damage. However, in prolonged and, even worse, chronic neuroinflammation, there is a continuous release of inflammatory mediators which can cause neuronal damage over time and major infiltration of peripheral immune cells, with a progressively destructive effect leading to neurodegenerative disease [37,38]. Hypoxia and ischemia are among the events triggering neuroinflammation [39]. Studies have highlighted the attention on the impact of chronic cerebral hypoperfusion (CCH) on the onset and progression of neurocognitive disorders such as Alzheimer’s disease (AD) [40,41]. Indeed, evidence suggests a connection between vascular risk and cognitive impairment [42]. Oxygen mediates a post-transcriptional modification on hypoxia-inducible factor-1 (HIF-1)-α and HIF-2α, triggering them to proteasome degradation [43,44]. In hypoxic conditions, this modification does not occur, leading to HIF1α and HIF2α stabilization and translocation to the nucleus where they mediate transcriptional regulation [45]. The regulated genes include those encoding enzymes involved in energy metabolism, apoptosis, and inflammation. Notably, adenosine receptors, enzymes responsible for adenosine metabolism (such as CD73 and CD39), and adenosine transporters are among them [46,47]. Among the main actors of neuroinflammation, we mention microglia and astrocytes.

Microglia cells are innate resident CNS immune phagocytic cells characterized by low turnover. Microglia cells exist in a homeostatic (surveying) state in healthy conditions, but become activated upon the perturbation of homeostasis, thus changing their morphological and protein expression patterns. Microglia express many pattern recognition receptors (PRRs) detecting pathogen-associated (PAMPs) or danger-associated (DAMPs) molecular patterns, including Toll-like receptors (TLRs) and their coreceptors, NOD-, LRR-, and pyrin domain-containing protein 3 (NLRP3) inflammasome, nucleic acids receptors, C-Type Lectin Domain-Containing 7A (CLEC7A), and others [48]. Inflammasomes are multimeric protein complexes that assemble in the cytoplasm after PAMPs and DAMPs recognition by PRRs. Inflammasomes thus recruit inactive pro-caspase-1 proteins whose oligomerization leads to their autoproteolytic cleavage, leading to active caspase-1, which, in turn, cleaves pro-IL-1β and pro-IL-18, generating IL-1β and IL-18. Currently, a two-signal model suggests that a first signal is required to prime NLRP3 and then a second signal is required to activate it. The first signal can consist of lipopolysaccharide (LPS) binding to TLR, leading to NF-kB activation and translocation into the nucleus, where it induces NLRP3 expression. Afterward, various stimuli, including ATP, pore-forming toxins, nucleic acids, hyaluronan, and fungal, bacterial, or viral pathogens, etc., induce NLRP3 formation [49,50].

Activation of microglia can be of the M1-type form through TLR and interferon γ (IFN-γ) signaling pathways, with microglia exerting a pro-inflammatory and neurotoxic role including the production of pro-inflammatory cytokines and chemokines, such as tumor necrosis α (TNF-α), interleukin 6 (IL-6), IL-1β, IL-12, and C-C motif chemokine ligand 2 (CCL2) and the expression of nitric oxidase and nicotinamide adenine dinucleotide phosphate (NADPH) oxidase, generating, respectively, nitric oxide (NO) and ROS.

The alternative form of activation, triggered by different stimuli, leads to M2 microglia, characterized by an opposite anti-inflammatory and neuroprotective effect, with the secretion of growth factors and anti-inflammatory cytokines such as IL-10 and transforming growth factor β (TGF-β) [48]. However, in vivo, there is no absolute separation between M1 and M2 active states, since different reactive phenotypes exist and appear to be context dependent [51].

Another CNS resident glial cell type playing a major role in neuroinflammation is astrocyte, which also distinguishes into A1 inflammatory and neurotoxic astrocyte and A2 neuroprotective astrocyte. Their activity is regulated by Janus kinase–signal transducer and transcription activator 3 (JAK/STAT3), nuclear factor kappa B (NF-κB), MAPK, and calcineurin pathways [52]. In homeostatic conditions, they also contribute to maintaining CNS homeostasis, while during neuroinflammation, they undergo a reactive state in which they participate in the release of pro-inflammatory cytokines. Moreover, astrocytes secrete various factors affecting tight junction proteins (e.g., morphogens such as Sonic hedgehog (Shh) and retinoic acid, growth factors such as vascular endothelial growth factor (VEGF) and many others), regulating the integrity and permeability of BBB, which can thus be altered once astrocytes are in the reactive state [53].

The alteration of BBB permeability let the migration of peripheral immune cells into CNS, such as neutrophils, which contribute to BBB impairment, monocytes, natural killer cells, dendritic cells, T cells, and B cells, with a consequent exacerbation of the neuroinflammation course [37].

### 1.3. Neurodegeneration

As previously stated, neuroinflammation, if not limited, can lead to CNS dysfunction and damage contributing to the complex multifactorial framework causing neurodegenerative diseases. Neurodegenerative diseases are characterized by the progressive dysfunction and loss of neurons in the CNS. Their mechanism is not completely clear, but it includes aging, immunity, and neuroinflammation [54].

Parkinson’s disease (PD), Alzheimer’s disease (AD), Huntington’s disease (HD), and Amyotrophic Lateral Sclerosis (ALS) are defined as neurodegenerative disorders whose features are neuronal dysfunction and death. All of them are characterized by common risk factors, such as oxidative stress, environmental factors, aging, and protein dysfunction. Regarding proteins, misfolding and aggregation of specific proteins is a common characteristic [55]. A brief introduction to the neurodegenerative disease under discussion in this review is provided in the following paragraphs.

#### 1.3.1. Alzheimer’s Disease

AD, the most common form of dementia, is characterized by synapse loss and neuronal atrophy that predominately hits the hippocampus first and then the cerebral cortex [56]. The disease leads to a progressive loss of memory, worsened cognitive functions including language, and failure in performing simple and daily activities [57,58]. The formation of extracellular senile plaques composed of amyloid β protein (Aβ) and intracellular neurofibrillary tangles (NFTs) consisting of hyperphosphorylated tau (τ) protein, are the typical hallmarks of the disease [59]. Aβ is generated through the proteolytic processing of amyloid-beta precursor protein (AβPP) by β-secretase 1 (BACE1) followed by γ-secretase [56]. AD is caused by a combination of both environmental and genetic factors; thus, it is a complex and multifactorial disease for which several hypotheses have been postulated, such as, along with amyloid and the tau protein, there are the cholinergic, inflammatory, glutamate excitotoxicity, oxidative stress, metal ion, microbiota–gut–brain axis, and abnormal autophagy [60]. The neurodegeneration characterized by neuronal loss is accompanied by the neuroinflammatory process, oxidative stress, and several cellular abnormalities [61]. Regarding neuroinflammation, both microglia and astrocytes are activated in AD. In the early stages, this activation is beneficial, as these glial cells help clear amyloid-beta (Aβ) deposits. However, as the disease progresses, chronically activated microglia begins to exert detrimental effects, contributing to neurodegeneration in the surrounding brain regions [62]. Astrocyte reactivity also intensifies with increasing Aβ accumulation and tends to co-localize with amyloid plaques. In contrast, astrocytes located away from plaques are often atrophic, leading to reduced support for neurons and synaptic connections. This loss of support is associated with early cognitive impairment [63].

#### 1.3.2. Parkinson’s Disease

PD is a progressive disorder of the central nervous system that affects mobility. The motor symptoms of PD are due to the degeneration of the dopaminergic neurons in the nigrostriatal pathway, which, in turn, resulted in the depletion of dopamine production [64]. The direct and indirect pathways of the basal ganglia are essential for motor control, both originating in the striatum and modulated by dopaminergic input. These pathways exert opposing effects on thalamic activity: the direct pathway facilitates movement by disinhibiting thalamic neurons, while the indirect pathway suppresses movement through increased inhibition. In Parkinson’s disease (PD), the degeneration of dopaminergic neurons in the substantia nigra pars compacta disrupts this balance, leading to underactivity of the direct pathway and overactivity of the indirect pathway. This imbalance contributes to hallmark motor symptoms such as bradykinesia, rigidity, and tremor [65,66]. Lewy Bodies are intracellular inclusions that represent one of the hallmarks of PD and are composed mainly of α-synuclein (α-Syn) aggregates, which the accumulation of contributes to the decrease in dopamine biosynthesis by the inhibition of tyrosine hydroxylase [67,68,69,70].

#### 1.3.3. Other Neurodegenerative Disease

Other neurodegenerative diseases involving ARs discussed in this review are ALS and HD.

ALS is characterized by the progressive loss and injury of motor neurons that leads to muscle weakness, followed by paralysis, and fatal respiratory failure. The main pathogenic factors of this disorder are represented by mutations in the genes of Trans activation response DNA-binding protein 43 (TDP-43), superoxide dismutase 1 (SOD1), Chromosome 9 Open Reading Frame 72 (C9orf72) protein, and Fused in Sarcoma (FUS, also called translocated in liposarcoma, TLP) protein [71]. These mutated proteins, and, in particular, TDP-43, are found in the aggregates typical of ALS [72]. Normally, TDP-43 is in the nucleus where it binds both DNA and RNA, regulating mRNA at different stages [73,74]. In ALS, TDP-43 mislocalizes from the nucleus to cytoplasm, leading to the deep deregulation of mRNAs in the cell. The trigger point that determines the degeneration of motor neurons is the aggregation of this anomalous protein in the cytoplasm within and outside stress granulates (SGs), contributing to the production of oxidative stress and neuronal toxicity [75,76,77,78]. In ALS, neuroinflammation involves both microglia and astrocytes, which acquire neurotoxic phenotypes that promote motor neuron death. Microglial activation has been observed early in disease progression, with elevated cytokines such as monocyte chemoattractant protein-1 (MCP-1) and IL-6 in the CNS and periphery. Astrocytic glutamate dysregulation and impaired support to neurons further exacerbate excitotoxicity [79].

Finally, HD is a hereditary condition marked by neuropsychiatric symptoms, a movement disorder—most commonly chorea—and progressive cognitive decline [80] characterized by progressive striatal neurodegeneration. The mutation responsible for this disease is an abnormally expanded and unstable CAG repeated within the gene-encoding huntingtin protein [81]. This is ubiquitously expressed, but high levels are present in the brain. Mutated huntingtin forms toxic mutant huntingtin oligomers. GABAergic medium spiny neurons of the striatum are the most vulnerable cells in HD [80].

In the following pages, the single AR subtypes are discussed separately, trying to summarize the main discoveries in terms of both general neuroinflammation and specific neurodegenerative diseases. In addition, at the beginning of every chapter, a list of the most common and significant ligands, both agonists and antagonists, used as tools for that specific receptor, is given with the affinity data for all the receptors when present, and, if available, also the data on the rat or mouse receptor, due to the usual employment of these species as animal models. The aim is to acknowledge the effective selectivity of the tools used, which can help to interpret data obtained from an experiment and create a guide to choose the correct tool for future experiments.

## 2. A_1_ AR

**Figure 3:**
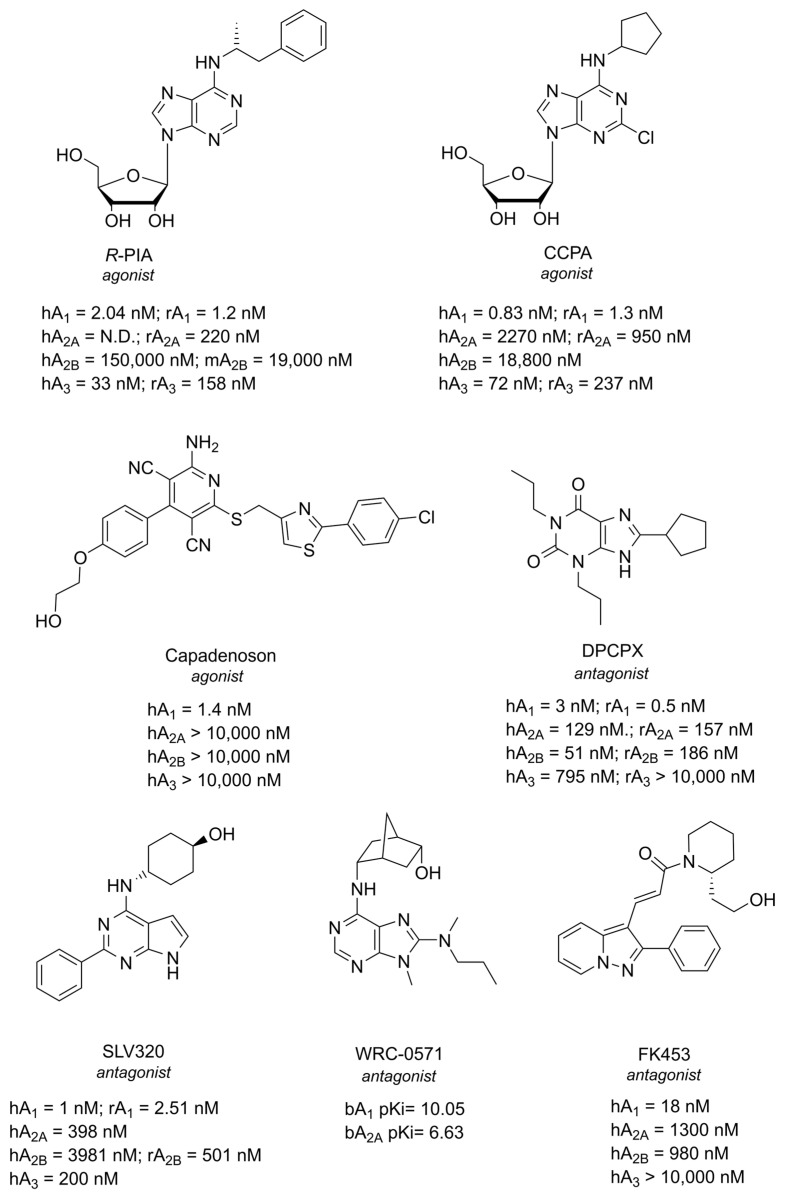
Structures of the most representative A_1_ AR ligands. All the reported values are Ki.

Being the first AR to be identified, most of the ligands for this receptor were discovered in the last century. The most representative agonists and antagonists used as pharmacological tools for A_1_ AR are reported in Figure 3, but it is important to highlight that several allosteric modulators have also been reported for this AR subtype [76,77,78,79].

### 2.1. Role in Neuroinflammation

**Figure 4:**
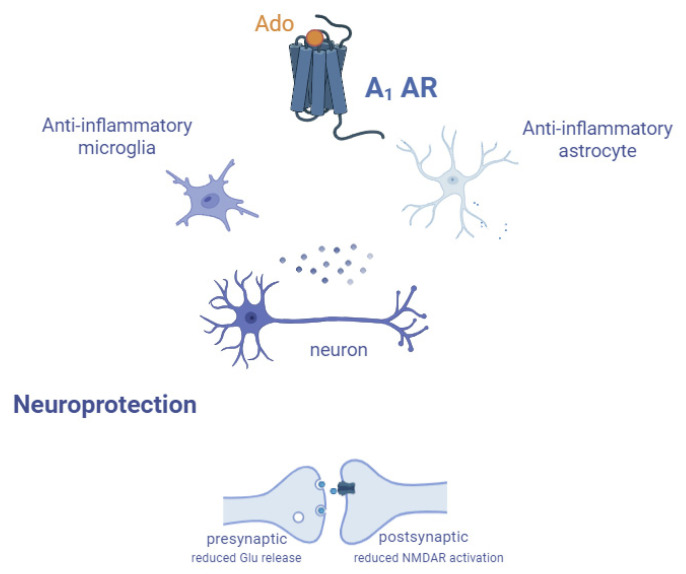
General neuroprotective effect mediated by A_1_ AR activation. Receptor activation has an anti-inflammatory effect on both microglia and astrocytes that, along with its ability to reduce glutamate (Glu) release at presynaptic level in neurons and to reduce postsynaptic NMDA receptor activation, confers global neuroprotection.

A_1_ AR is widely expressed in the brain and particularly in neurons and microglia [15,82]. Even if microglia activation can be considered the initial trigger of neuroinflammation [83], the activation of A_1_ AR in these cells was found to both promote and counteract inflammation [84,85,86,87]. Diseased A_1_ AR knockout (KO) mice (e.g., by traumatic brain injury, TBI [88], or glioblastoma [89]) showed an increased activity of microglia and neuroinflammation, suggesting an anti-inflammatory role for the A_1_ AR (Figure 4). Chronic caffeine intake, which causes A_1_ AR upregulation, alleviates inflammation in a model of multiple sclerosis (MS) [86]. A_1_ AR agonists were found to inhibit the morphological activation of microglia, probably blocking the calcium influx triggered by ATP treatment [90].

Astrocytes express all ARs on their membrane but A_1_ ARs reveal the highest binding ability to adenosine, which leads to a decrease in the production of IL-12, IL-23, and chemokines, generating an immunosuppressive effect useful in conditions like autoimmune encephalomyelitis (Figure 4) [91]. On the contrary, recently, Guo et al. demonstrated that A_1_ ARs in astrocytes, and not in pericytes, microglia, or oligodendrocyte precursors cells, trigger the early stage of the inflammatory response of microglia in a model of LPS-induced sepsis-associated encephalopathy (SAE) [26]. Another crucial point highlighted by this work is that peripheral adenosine could pass the BBB, directly enhancing its central extracellular levels [92].

It is worth noting that adenosine-A_1_ AR and glutamate signaling are strictly connected, partly explaining the adenosine-mediated neuroprotective effect [5]. In fact, presynaptic A_1_ AR decreases glutamate release in synapses, while post-synaptic A_1_ AR activation limits N-methyl-D-aspartate (NMDA) receptor activation and calcium current influx, suppressing excitatory action potentials (Figure 4) [84,93]. Under hypoxic conditions, this response is thought to serve as a compensatory strategy to safeguard neurons by balancing the oxygen supply and its consumption [94]. In seizures, conditions characterized by neuronal hyperexcitability, A_1_ AR was found to be neuroprotective [83]. Regarding the time course of its neuroprotective effect, adenosine has been shown to inhibit excitatory synaptic transmission in the hippocampus, probably via A_1_ AR, both during ischemia and for a significant period afterward [4].

The relation between adenosine and neuroinflammation has also been investigated in depression, a psychiatric disorder. Adenosine has been found to behave with antidepressant effects, especially towards A_2A_ AR, discussed afterwards, but also by A_1_ AR activation by astrocyte-derived adenosine [85,95,96].

As reported in the introductory part, A_1_ AR is expressed in heart tissue, where the use of agonists can cause bradycardia, atrioventricular block, or hemodynamic effects. Possible strategies to avoid this include using partial agonists, positive allosteric modulators, or biased agonists instead of full agonists [97,98].

### 2.2. Role in Neurodegenerative Disorders

There are several theories regarding the mechanisms of neurodegeneration. One of these is the glutamate hypothesis, which postulates that neurodegeneration is driven by excitotoxicity resulting from excessive glutamate signaling. Excessive recruitment of NMDA receptors, which is associated with spreading depolarization, leads to dysregulated calcium influx, ultimately causing neuronal damage [99]. As reported before, adenosine, mainly through A_1_ AR, decreases this excitatory transmission [93,100], and several studies reported a related decrease in the neurodegenerative process both in in vitro and in vivo models [101,102,103,104,105,106]. In fact, as already highlighted, adenosine, also mainly through A_1_ AR, affects metabolism activity not only in neurons but also in astrocytes and microglia [47,107,108,109,110,111]. These findings contrast with the documented neuroprotective effect attributed to the AR antagonist caffeine when consumed in a chronic but moderate way [112], even if it has been related to an A_1_ AR upregulation [113].

The principal drawback in using full A_1_ AR agonists to treat neurodegenerative diseases is the on-target side effects on the heart [98]. In addition, A_1_ AR agonism has been reported to be protective in several non-central tissues, suggesting a general protective mechanism that is not solely related to its role as a neuromodulator [114]. Another important aspect to consider when evaluating A_1_ AR as a target in neurodegenerative diseases is that neuroprotection has only been observed when receptor activation occurs shortly before or simultaneously with the noxious stimulus. During such events, elevated levels of extracellular adenosine led to A_1_ AR desensitization, resulting in a loss of neuroprotective effects and, in some cases, even worsening brain damage after prolonged exposure to adenosine [93,115,116,117]. This observation aligns with recent studies on AR KO mice, which suggest that A_1_ AR primarily lays a role in the early stages of neuronal damage [106]. This supports the idea that A_1_ AR activation may have a more prophylactic than therapeutic effect. Additionally, adenosine released at the site of injury can diffuse to nearby regions, providing localized protection—a phenomenon known as brain preconditioning [118,119]. Therefore, A_1_ AR agonism may be beneficial in cases of acute, repetitive, and short-term brain insults. However, in the context of chronic neurodegenerative processes, dysfunctional astrocytes lose their ability to mediate preconditioning, contributing to the spread of neurodegeneration [120].

#### 2.2.1. Alzheimer’s Disease

It is important to note that the role of ARs in Alzheimer’s disease is under-investigated compared to Parkinson’s disease and has been studied primarily in in vitro and in vivo models. However, post-mortem brain samples from AD patients showed a decreased expression of A_1_ ARs in dentate gyrus, CA1 and CA3 regions of the hippocampus, which was attributed to neuronal loss in these brain regions, the most involved in learning and memory [121,122,123,124]; but a reduction of A_1_ ARs in the CA1 region of the hippocampus has also been observed in other disorders with dementia [125]. Instead, Angulo et al. found a non-significative difference in mRNA levels of A_1_ AR in the hippocampus of AD patients with respect to healthy subjects, but they found an increase in protein levels in degenerating neurons with NFT and in the dystrophic neurites of senile plaques [126]. Concerning senile plaques, in human neuroblastoma cells, A_1_ AR activation increased the production of a soluble secreted form of APP, supporting the use of agonists for the treatment of AD [126,127,128]. Another more recent study reported that A_1_ AR, along with A_2A_ AR, showed increased expression in the frontal cortex in AD [129].

#### 2.2.2. Parkinson’s Disease

A_1_ AR is highly expressed in substantia nigra (SN), and its gene is in a locus strongly related to PD [130]. Mutation or disfunction of A_1_ ARs are related to a worse scenario of the disease, inhibiting dopaminergic signaling [130,131,132]. In fact, A_1_ AR co-localizes with the dopamine D1 receptors on the GABA/dynorphin output neurons, where they can influence each other’s function at either the receptor or intracellular signaling cascade level [133]. A_1_ AR/D1 R can form functional heteromers, where A_1_ AR antagonistically and specifically modulate the binding and functional characteristics of dopamine D1 receptors [133,134,135,136]. Instead, A_2A_ ARs are mostly highly expressed in the intermediate spiny neurons of the striatum in close association with dopamine D2 receptors (indirect pathway) and, again, specific negative interactions have been described [133,134,137].

As already said, chronic A_1_ AR stimulation may aggravate neuroinflammation and, in PD, has also been observed to induce synucleinopathy in some rodent models [138,139]. The mechanism seems to reside in the binding of the A_1_ AR to the N-terminus of α-syn, promoting its misfolding [113,139]; this suggests that not only A_2A_ AR is involved in this pathology and that further studies are needed to understand the exact role of this receptor in the disease.

## 3. A_2A_ AR

**Figure 5:**
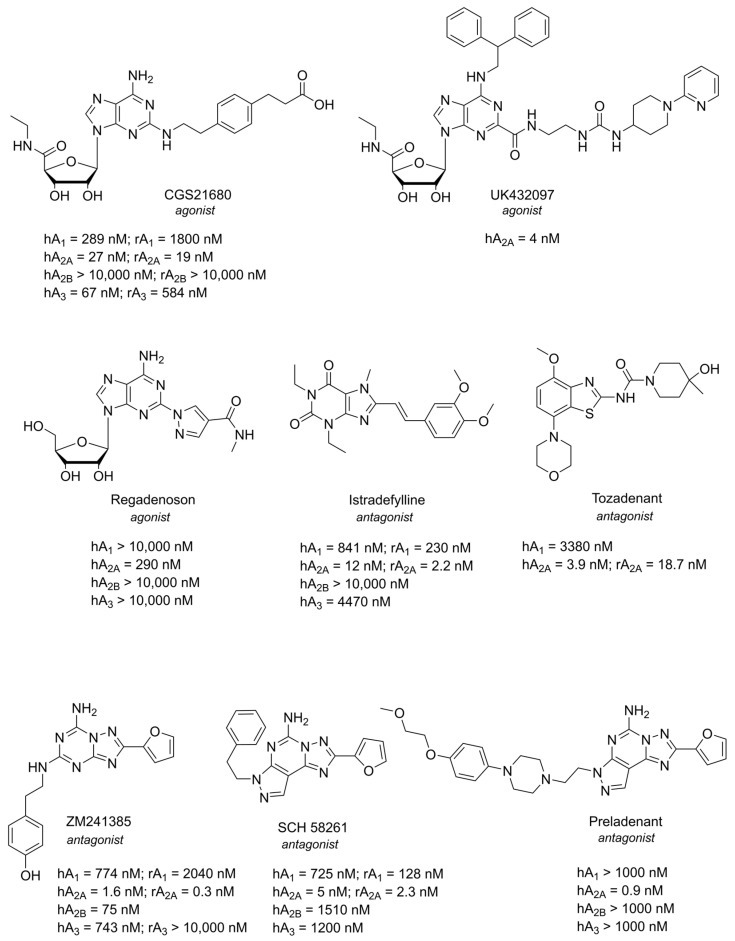
Structures of the most representative A_2A_ AR ligands. All the reported values are Ki.

A_2A_ AR has been extensively studied, and a huge number of selective agonists and antagonists are available. A considerable number of them reached or are still in clinical trials, in particular, for the treatment of Parkinson’s disease and cancer. Among agonists: Regadenoson is an A_2A_ AR agonist approved for myocardial perfusion imaging, while Istradefylline is an approved antagonist as adjuvant in classical dopaminergic PD therapy. Representative ligands are reported in Figure 5 [15,140,141].

### 3.1. Role in Neuroinflammation

**Figure 6:**
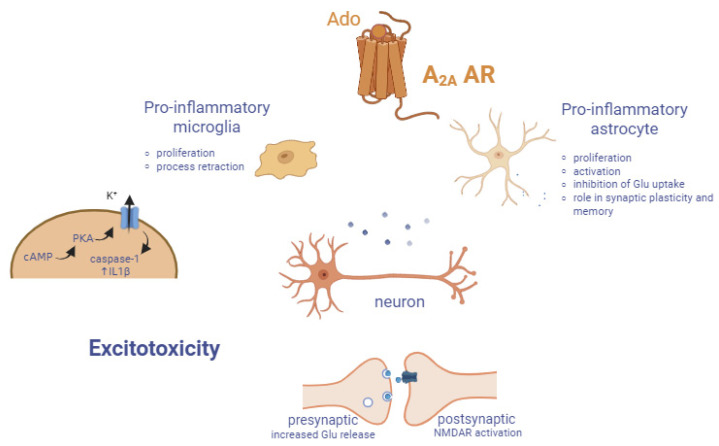
General pro-inflammatory and excitotoxic effects mediated by A_2A_ AR activation. Receptor activation had a pro-inflammatory effect on both microglia and astrocytes. In addition, A_2A_ AR activates PKA via cAMP, which enhances potassium efflux and subsequently triggers caspase-1 activation, leading to increased levels of the pro-inflammatory cytokine IL-1β. In neurons, presynaptic A_2A_ AR activation increases glutamate (Glu) release, participating in the excitotoxic effect.

In general, adenosine receptors, especially towards A_2A_ AR, are involved in inflammasome activation; in fact, the increase in cAMP levels is followed by PKA activation, which leads to an augmented potassium efflux, which triggers caspase-1 activation [142,143]. Moreover, elevated adenosine levels in the extracellular space have been associated not only with increased caspase-1 activity but also with enhanced IL-1β production (Figure 6) [142,144]. As for A_1_ AR, A_2A_ AR also regulates both microglia and astrocytes during neuroinflammatory events, and, again, showing both pro- and anti-inflammatory effects [83,145]. In particular, Pedata et al. reviewed the role of A_2A_ ARs in brain ischemia, suggesting that antagonists for these receptors are protective during acute injury by alleviating hyper-excitotoxicity at a central level, while agonists allow prolonged protection by regulating leukocyte infiltration for days after the ischemic event [146]. The sustained anti-inflammatory behavior of A_2A_ AR agonists was also observed after blunt spinal trauma, resulting in a reduction in long-term neurologic injury [147].

As reported earlier in this review, adenosine, via A_2A_ AR, mediated vasodilation; in fact, the agonist Regadenoson is clinically used in imaging with this purpose. Thus, the use of A_2A_ AR agonists for their anti-inflammatory (or other) effect is accompanied by vasodilation and a decrease in blood pressure, which should be taken into consideration for drug development purposes. The role of cardiac A_2A_ AR has been quite recently reviewed [148,149].

In particular, A_2A_ AR was found to have a role in hyperexcitability and excitotoxicity (i.e., by increasing glutamate release) in the CNS, not only in the striatum but also in the hippocampus and cortex (Figure 6) [150,151,152]. The other big role of A_2A_ AR upregulation is that of control over microglial activity (Figure 6) by (1) promoting their proliferation and (2) mediating microglial process retraction, an important marker of neuroinflammation [152,153,154,155]. In physiological conditions, A_2A_ AR is not highly expressed on microglia, but its expression increases as a result of brain insults leading to the activation of signaling pathways different to those active in the physiological state [156]. Process retraction conveys a modification of microglia cells from a ramified to an amoeboid shape, and, specifically, A_2A_ AR mediates activation to the so-called M1 phenotype, which is characterized by the overexpression of inflammatory enzymes and the release of pro-inflammatory mediators [153,157,158]. A_2A_ AR antagonists seem to be beneficial in animal models of perinatal brain injury and of Parkinson’s disease [155,159]. Similar behavior has been observed in anxiety disorders, which are also characterized by altered microglia. These disorders can be caused by prenatal exposure to glucocorticoids, and perinatal treatment with A_2A_ AR antagonists was able to ameliorate the inflammatory state and anxiety behavior [87,144].

Even if the principal effects of A_2A_ AR activation are observed in microglia, there are also some studies on astrocytes. This receptor regulates the uptake of both GABA and glutamate, thus fine-tuning the inhibitory and excitatory modulation at synapses [160,161]. A_2A_ AR antagonists were able to inhibit astrogliosis both in vitro and in vivo, contributing to understanding the mechanism of A_2A_ AR blockade-mediated neuroprotection (Figure 6) [162,163]. Due to the A_2A_ AR upregulation observed in AD, A_2A_ AR overexpression in primary cortical astrocytes has been studied, revealing alterations in the astrocytic transcriptome with an important effect on genes relevant for cell activation, immune response, and also angiogenesis [164]. Various studies concur in attributing a significant role to astrocytic A_2A_ AR in regulating synaptic plasticity and memory, thereby opening new avenues for therapeutic interventions targeting this receptor (see discussion below) [63,160,165]. It should be noted that opposite results were instead reported by Yuan et al. in mice with CCH-induced white matter lesions, in which authors observed an anti-inflammatory effect by A_2A_ AR activation, which involves STAT3/YKL-40 inhibition on astrocytes [166]. In a model of oxygen–glucose-deprived (OGD) hippocampal slices, A_2A_ ARs are protective by delaying astrocyte activation along with the appearance of anoxic depolarization [167]. This is in line with the well-known role of A_2A_ ARs in cancer, which are involved in the tumor immune escape [168].

Finally, as anticipated, the antidepressant effect of adenosine is mainly attributed to its action through the A_2A_ AR [85,95,96], even if A_2A_ AR KO or the use of antagonists, including caffeine, also demonstrated antidepressant-like effects in in vivo tests specific to predict antidepressant activity [85,169,170]. This effect is principally due to the counterbalance given by A_2A_ AR to the reduction in dopamine via A_2A_ AR-D2 R heteromer regulation [169].

### 3.2. Role in Neurodegenerative Disorders

A_2A_ ARs, due to their ability to bind SAP102 (synapse-associated protein 102), are mainly located in glutamatergic synapses [171], where they activate neurons in the hippocampus. Specifically, A_2A_ AR activation increases the entry of calcium through voltage-sensitive calcium channels, depolarizes neurons, and enhances the release of glutamate and the activity of NMDA receptors, thus promoting excitotoxicity [115]. The use of A_2A_ AR antagonists arose as an efficient therapeutic strategy in various models of neurodegenerative disorders [172]. Then, the role of A_2A_ ARs already discussed on glia cells supported this receptor as a valuable target in neurodegeneration [173]. In addition, a correlation between A_2A_ AR expression in the brain with those in platelets indicated the receptor as a possible marker of AD [174].

The use of A_2A_ AR antagonists for neurodegenerative diseases could lead to side effects related to the other phenomena mediated by receptors. In particular, an antagonist could potentially induce vasoconstriction, inflammation, or sleep disturbance. Of note, the FDA approved Istradefyllinedoes not exhibit these side-effects. Reasonably, the use of A_2A_ AR antagonists should be safe. Some other side effects that resulted in failed clinical trials for other compounds (i.e., Tozadenant for agranulocytosis) were not clearly proven to be on-target side effects [175].

#### 3.2.1. Alzheimer’s Disease

In the post-mortem brains of AD patients, both A_1_ and A_2A_ AR levels were increased in the frontal cortex [128,129]. In healthy subjects, A_2A_ AR is mainly present in striatal neurons, while in post-mortem AD samples, it has been primarily found in glial cells, not only in the cortex but also in the hippocampus [126,176]. Thus, combining these data, along with that already reported concerning the A_1_ AR, it can be pointed out that the expression and distribution of ARs in the AD are region and cell specific. In particular, A_2A_ AR in AD is highly expressed in astrocytes, where it regulates Na^+^/K^+^-ATPase, responsible for sustaining several processes occurring at the membrane level, like the regulation of glutamate transporter 1 (GLT-1), which controls glutamate uptake [177]. A_2A_ AR genetic removal in astrocytes enhances the memory performance of aged mice [178]. Besides the general neuroprotective effect observed with caffeine or the use of A_2A_ AR antagonists, caffeine also demonstrated a beneficial effect in specific transgenic mouse models of AD-bearing mutant forms of AβPP and/or presenilin-1 (PS1) [179,180,181], reducing both synapsis loss and neurotoxicity in the hippocampus of rat models [182]. All these findings indicated a crucial role of A_2A_ AR in controlling synaptic plasticity and memory, thus suggesting that antagonists are useful to prevent synaptic dysfunction and cognitive deficits like memory loss [183,184,185]. Temido-Ferreira et al. found that the overexpression of A_2A_ AR in rats is sufficient to impair the LTD-to-LTP transition in neurons, a hallmark of memory impairment [186].

Recent studies provide compelling evidence that neuronal A_2A_ ARs dysfunction, as seen in the brains of patients, contributes to amyloid-related pathogenesis and underscores the potential of A_2A_ AR as a relevant therapeutic target for mitigating cognitive impairments in this neurodegenerative disorder [187]. In addition, the impact of astrocytic A_2A_ AR upregulation has been highlighted very recently, as seen in various neurological conditions, on the development of a detrimental multicellular response associated with memory alterations and provides an additional proof-of-concept for the value of targeting this receptor in different neurodegenerative conditions [188].

#### 3.2.2. Parkinson’s Disease

A_2A_ ARs play a crucial role in the pathophysiology of PD due to their interaction with the dopaminergic system in extrapyramidal regions. Specifically, A_2A_ AR antagonists are therapeutically beneficial by reducing the inhibitory output of the basal ganglia’s indirect pathway, thereby helping to restore motor function [128,189,190]. In fact, in the cell bodies of medium spiny neurons in the striatum, A_2A_ AR and D2 Rs are co-localized and interact antagonistically. This existence of A_2A_-D_2_ heteromeric complexes has been demonstrated on co-immunoprecipitation studies and on fluorescence resonance energy transfer and bioluminescence resonance energy transfer analyses. It has now become possible to show that A_2A_ and D_2_ receptors also co-immunoprecipitate in striatal tissue, giving evidence for the existence of A_2A_-D_2_ heteromeric receptor complexes also in rat striatal tissue [191].

Thus, the blockade of A_2A_ AR enforces the effect of the diminished D2R-induced stimulation by pathologically low dopaminergic innervation from the damaged substantia nigra pars compacta. Reported effects of blocking A_2A_ AR in PD are the improvement of motor function, attenuation of dyskinesia, reduction in α-synuclein aggregation, and alleviation of non-motor symptoms like depression and cognitive disfunction [192,193,194,195,196,197]. In addition, as already mentioned in the neuroinflammation section, there is a high level of activation of microglia in the midbrain/substantia nigra in PD, and A_2A_ AR antagonists are found to return microglia in their resting state, decreasing the neuroinflammatory response [155,198]. It is important to note that A_2A_ AR antagonists are effective in PD but as an adjuvant therapy to complement dopamine D2 R agonists treatment [199]. Several A_2A_ AR antagonists are in human trials for Parkinson’s disease based on their motor enhancement feature in various animal models; nevertheless, only a compound named Istradefylline (1,3-diethyl-8-(3-methoxystyryl)-7-methilxanthine has been approved in Japan and the USA for treatment of basal ganglia disorders such as Parkinson’s disease, in association with L-DOPA [193,200]. Several studies on xanthine-based A_2A_ AR antagonists for the treatment of PD have been reported so far; Petzer at al. investigated if some of these compounds, including Istradefylline, were active also on monoamine oxidase B (MAO-B). MAO-B is the major enzyme responsible for the dopamine catabolism in CNS; thus, its inhibition is another useful strategy in PD therapy. The results showed that most compounds inhibited MAO-B in the micromolar range compared to their nanomolar affinity for the A_2A_ AR. This suggests that both in vitro and in vivo findings should be interpreted with caution, as the observed effects may result from multiple mechanisms of action. Results demonstrated that most of them inhibited MAO-B in the micromolar range (respect to nM affinity at the A_2A_ AR), revealing that results obtained in vitro or in vivo should be cautiously interpreted because they can be ascribed to a multiple mechanism of action. It is important to note that several A_2A_/MAO-B dual-targeting ligands were rationally designed in recent decades [201,202,203,204].

#### 3.2.3. Huntington’s Disease

In HD, A_2A_ AR is highly expressed in the vulnerable GABAergic enkephalin neurons of the basal ganglia but not in other neurons that equally express mutant and normal huntingtin, suggesting an involvement of A_2A_ ARs in HD [205]. Experimental models of HD revealed alterations in the expression of A_2A_ AR as also in their signaling, which has also been observed in peripheral blood cells of HD patients. This higher expression in mouse models has been related more from an altered receptor turnover than from an increased transcription, as A_2A_ AR mRNA levels remain unchanged [206,207]. In a mouse model of HD, a specific altered A_2A_ AR phenotype has been found in the early stages of disease [207]. Instead, in later stages, a significant decrease in A_2A_ AR transcription has been observed. However, conflicting data about the potential neuroprotective and neurodegenerative effects of these receptors in the brain have been reported, with beneficial effects shown by both A_2A_ AR agonists and antagonists [205,208].

#### 3.2.4. Amyotrophic Lateral Sclerosis

A_2A_ AR antagonists have been reported to reduce motoneuron death in ALS [209,210,211]. The proposed mechanism is that motoneurons’ vulnerability to excitotoxicity is due to the tyrosine receptor kinase B (TrkB) activation by the brain-derived neurotrophic factor (BDNF) and A_2A_ AR transactivates TrkB. This transactivation occurs even in the absence of the ligand BDNF; it is BDNF-independent [209,210]. On the contrary, another research group advanced an opposite theory based on the fact that neurotrophins are potent survival factors for developing and injured neurons and that neurotrophins were not useful for therapy due to administration and side effect issues. Thus, they suggest using transactivation, like that mediated by A_2A_ AR for TrKB, to trigger a neuroprotective effect [177]. In fact, in their work A_2A_ AR agonists were able to delay disease onset in mice [177]. Authors also reported the inability of BDNF to protect motoneurons and attributed this to the overactivation of a truncated form of the TrkB receptor rather than the full-length form transactivated by A_2A_ AR agonists [212,213]. In SOD1(G93A) mice, the classical animal model of ALS, which bears a mutation in the superoxide dismutase 1 (SOD1) gene, the effect of A_2A_ AR agonists has been investigated both in the pre-symptomatic and symptomatic stage of the disease (e.g., 4–6 weeks and 12–14 weeks old, respectively). Results indicated a beneficial involvement of A_2A_ AR only in pre-symptomatic individuals, which is in line with the previous evidence discussed of the A_2A_ agonist-delaying effect on the onset of ALS [214]. The symptomatic stage has also been related with the impairment of long-term potentiation (LTP), suggesting that the decreased synaptic plasticity can involve the A_2A_ AR over-activation of the early disease stages [215]. In another ALS mouse model, which expressed human TDP-43, A_2A_ AR activation rescued TDP-43 cytoplasm mislocalization, which has been proven to be counteracted by the activation of the co-localized D2R in the motor neurons of ALS patients [216,217].

Regarding disease progression, in experiments where pharmacological inhibition and the partial genetic ablation of A_2A_ ARs were performed in SOD1(G93A) mice, a significant delay in disease progression was observed. A study by Armida et al. aimed to clarify existing uncertainties by administering an A_2A_ receptor agonist or antagonist to mice starting from the presymptomatic stage. Unfortunately, in neither case was there an improvement in motor skill deterioration or survival in SOD1(G93A) mice [218].

Conversely, in the same animal model treated with a daily intake of caffeine for 70 days, mice experienced a significantly shortened survival [219].

In a study performed on 377 newly diagnosed ALS patients compared to 754 controls, caffeine was found to reduce ALS risk, while in another study on a larger cohort of patients, this association has not been demonstrated [220,221]. A recent study aimed to investigate the effect of caffeine consumption on ALS patients failed to find a correlation with disease progression and survival, but higher caffeine consumption was found to significantly improve the cognitive performance in a specific cohort of patients that carry the minor allele T of rs2472297 and are considered as fast metabolizers [222]. Thus, high discrepancy on the role of A_2A_ AR in ALS is present due to the complex role of the purinergic signaling in a multifactorial and multisystemic disease like ALS [128]. Vincenzi et al. showed the upregulation of A_2A_ AR in lymphocytes from blood samples of ALS patients, demonstrating a positive correlation between receptor density and the scores of the Revised Amyotrophic Lateral Sclerosis Functional Rating Scale (ALSFRS-R) that suggest, again, a possible protective effect of this receptor subtype [223].

#### 3.2.5. Others

As reviewed by Cunha R. A., A_2A_ AR antagonists have also been proposed for other neurodegenerative disorders like diabetic neuropathy, ataxia-related Machado–Joseph disease, experimental autoimmune encephalomyelitis, and subarachnoid hemorrhage [115].

Pharmacological studies by Chen et al. indicate that A_2A_ ARs play a prominent role in the development of ischemic injury within the brain and demonstrate the potential for anatomical and functional neuroprotection against stroke by A_2A_ receptor antagonists [224].

## 4. A_2B_ AR

**Figure 7:**
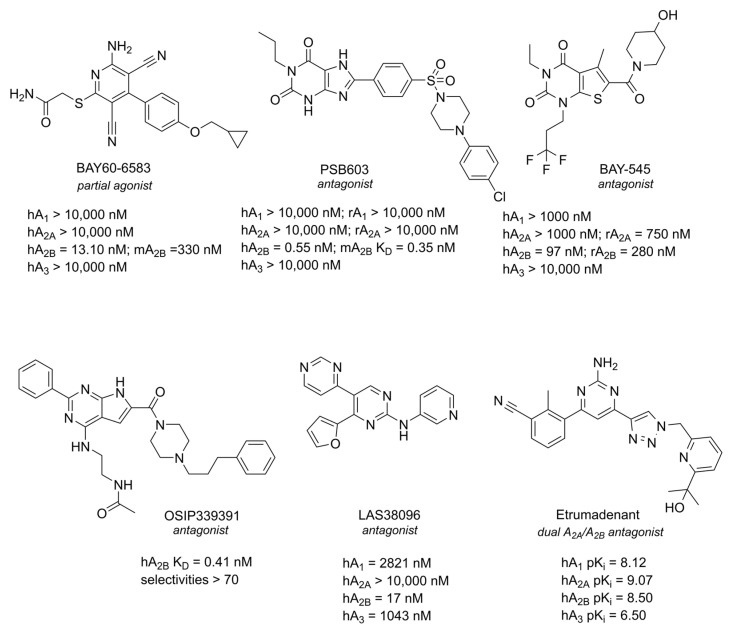
Structures of the most representative A_2B_ AR ligands. All values reported are Kis, unless stated otherwise.

A_2B_ AR, which is activated only at high concentrations of adenosine, has been a challenging target for the development of selective ligands. In fact, only in recent decades have effective pharmacological tools become available. To date, no potent and selective A_2B_ AR agonists are accessible. BAY60-6583, which was initially defined as an agonist, has since been shown to behave as a partial agonist [225]. Instead, several xanthines have initially been reported as selective antagonists (i.e., PSB603 and the xanthine-like BAY-545), but as already said, recently, new structures have been developed (i.e., OSIP339391 and LAS38096) [15,17,226,227]. Representative structures of ligands towards A_2B_ AR are displayed in Figure 7. Etrumadenant is a dual A_2A_ AR/A_2B_ AR antagonist currently in clinical trials for cancer [228,229] but is reported here as an example of a dual-targeting ligand that, due to the complex regulation in the CNS by different AR subtypes, could be an effective strategy for the treatment of neurodegenerative diseases.

### 4.1. Role in Neuroinflammation

A_2B_ AR are widely distributed throughout the brain [230]. In primary murine microglia, through the p38 MAPK pathway, the A_2B_ AR has been found to promote the production of both anti-inflammatory cytokine IL-10 [231] and pro-inflammatory IL-6, along with the stimulation of microglia proliferation [232,233]. Downregulation of A_2B_ AR in inflamed brain is reported [234].

A_2B_ AR has been reported among the most highly expressed G protein-coupled receptors in astrocytes [235], and it was found to mediate their neuronal activity-dependent metabolic activation and a consequent involvement in sleep and memory. Some neurological diseases are characterized by an excess in excitatory synapses, and A_2B_ AR reduces their number in postnatal development by a decrease in metabotropic glutamate receptor 5 (mGlu5) expression in astrocytes [236]. On the other hand, A_2B_ AR antagonists were found to be protective in hippocampus subjected to severe hypoxic/ischemic conditions, but they were not able to counteract glutamate-induced injury, thus revealing a mechanism of action upstream to glutamate release [237]. Coppi et al. reviewed the role of A_2B_ AR in brain ischemia and subsequent demyelination injury. It is located on any cell type of the brain and on vascular and blood cells participating in both the salvage and damage of the tissue, including protracted demyelination [238]. These findings point out A_2B_ AR as a possible target in neurodegenerative and neurological diseases. Its low affinity for adenosine makes it particularly intriguing, as it becomes activated only under pathological conditions characterized by elevated extracellular adenosine levels—offering a degree of selectivity in targeting disease states [239].

Thus, they all represent important targets for drugs having different therapeutic time-windows after stroke. The final protective outcome for an agonist versus antagonist compound depends on the time of administration and district of activation of the receptor targeted by the drug.

Recently, because the literature points out that selective antagonism at both A_2A_ AR and A_2B_ AR delays the anoxic depolarization induced by a severe OGD insult in the hippocampus, Venturini et al. investigated a dual A_2A_/A_2B_ AR antagonist, P626, which was shown to be effectively neuroprotective [240]. Later, Dettori et al., used another dual antagonist, MRS3997, on a different model, the rat model of transient middle cerebral artery occlusion (tMCAo), obtaining a reduction in brain ischemic damage and neuroinflammation acting at both astrocytes and microglia, thus combining the neuroprotective effect of both A_2A_ ARs and A_2B_ ARs [241].

### 4.2. Role in Neurodegenerative Disorders

There are few articles concerning the role of A_2B_ AR and A_3_ AR in neurodegenerative disease. Concerning A_2B_ AR in mice treated with Aβ to infer a cognitive deficit, A_2B_ AR has been found to be downregulated along with the impairment of mitochondrial activity that the same authors reported to be rescued by treatment with an A_2B_ AR agonist. Of note, the agonist used is NECA, which is a well-known pan-agonist for ARs [242,243]. One interesting involvement of A_2B_ AR in neurodegenerative diseases comes from the leaky gut, one of the syndromes related to AD and also PD [244]. Recently, Ishioh et al. showed that intestinal permeability is regulated through basal forebrain cholinergic neurons (BFCN) mediated by A_2B_ AR and the vagal pathway [245,246,247]. The same group further investigated the signaling pathway, finding a key role for brain H_1_ receptor signaling [248]. These findings pave the way for new targets to be considered in neurodegenerative diseases, especially for AD.

## 5. A_3_ AR

**Figure 8:**
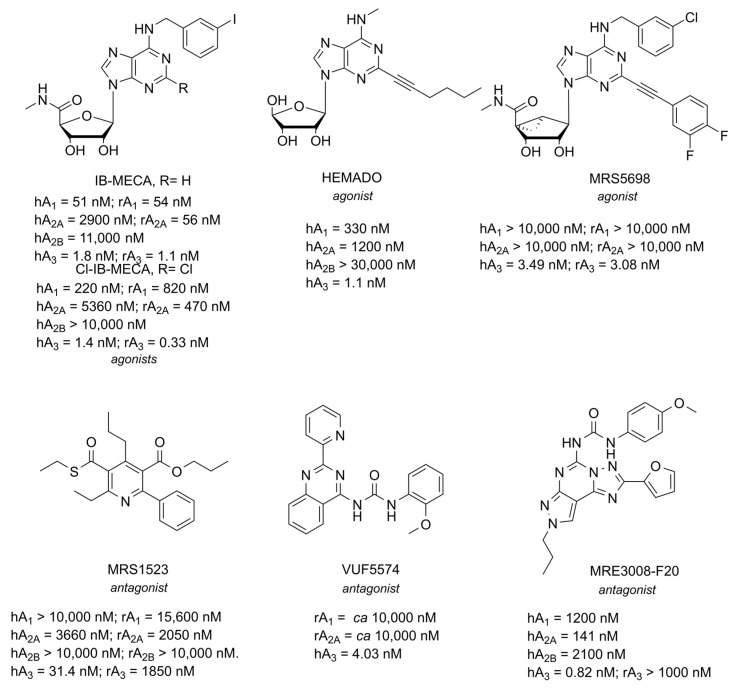
Structures of the most representative A_3_ AR ligands.

The most representative pharmacological tools for the A_3_ AR subtype are reported in Figure 8 [15,249,250]. Up to now, no A_3_ AR ligand has reached the market, but agonists IB-MECA and Cl-IB-MECA are under clinical investigations for cancer and autoimmune diseases [140]. The major challenge in investigating the A_3_ AR lies in the significant interspecies differences between the receptor in standard animal models, such as rats and mice, and in humans. Jacobson addressed this issue by developing MRS5698, a compound that is equipotent at both the mouse and human A_3_ AR [251]. This makes it a valuable tool for studying the role of A_3_ AR at the preclinical level [252]. Concerning A_3_ AR antagonists, a huge number of potent and selective compounds have been developed [20], exploring quite a wide chemical space: MRS1523, VUF5574, and MRE3008-F20, frequently used as pharmacological tools for this receptor, with MRS1523 also displaying an affinity against mouse and rat A_3_ AR, while the other two are completely inactive towards rodent receptors [253].

### 5.1. Role in Neuroinflammation

With respect to A_1_ AR and A_2A_ AR, there are few studies concerning A_3_ AR involvement in neuroinflammation. Although most studies attribute microglial process retraction to A_2A_ AR activation [152], A_3_ AR was also reported as a mediator of this process [254]. In contrast, Choi et al. showed that A_3_ AR can reduce inflammatory cell migration, including that of microglia, during brain ischemic injury [255]. Furthermore, one of the mechanisms underlying the A_3_ AR-mediated attenuation of neuropathic pain involves the inhibition of excessive microglial activation in the spinal cord dorsal horn [256,257]. A recent transcriptomic study investigated the effects of A_3_ AR agonists on gene regulation in activated microglia. Although the authors identified several genes whose expression was altered by A_3_ AR agonists, only a few showed statistically significant changes. Moreover, it remains difficult to attribute these changes to the receptor’s ability to mediate a transition between distinct microglial phenotypes. Therefore, further experiments are necessary to clarify these findings [258]. Repeated episodes of hypoxia on A_3_ AR KO mice led to a major vulnerability of hippocampal pyramidal neurons with respect to A_3_ AR^+/+^ mice, resulting in a decline in cognitive function [259]. Pugliese et al. investigated the role of A_3_ AR in rat hippocampal slices under severe ischemic conditions. Treatment with antagonists or with an agonist, but for a long time allowing receptor desensitization, led to a neuroprotective effect [260,261]. The group of Salvemini D. provides support for the beneficial effects of A_3_ AR agonists to mitigate secondary tissue injury and cognitive impairment following TBI [262].

The A_2A_ AR and A_3_ AR interact to form heteromeric complexes, of which the expression is higher in neurons from the striatum than from the cortex or hippocampus, and it is similar in activated or resting microglia, but it has been found upregulated in an AD model. Between them, there is a negative functional cross-talk, thus A_2A_ AR antagonists make A_3_ AR responsive to endogenous adenosine, reinforcing the therapeutic interest of A_2A_ AR antagonists to fight AD [263].

### 5.2. Role in Neurodegenerative Disorders

A specific role of A_3_ AR in AD has been ascribed in relation to the generation of intracellular Aβ. The mechanism involves the cell internalization of the precursor AβPP, with caffeine able to decrease AβPP internalization and, consequently, lower intracellular levels of Aβ. The effect of caffeine has been replicated using a selective A_3_ AR antagonist and not by A_1_, A_2A_, or A_2B_ ARs antagonists, suggesting that it is the A_3_ AR the subtype involved [264].

## 6. Conclusions

Adenosine receptors play a complex and not yet fully understood role in neuroinflammatory and neurodegenerative processes. Among them, A_1_ ARs and A_2A_ ARs are the most prominently involved, affecting both neuronal and glial cells. The intricate cross-talk between these cell types varies not only spatially but also temporally, making it challenging to determine whether agonists or antagonists are more appropriate, and whether a single-target or multitarget approach should be pursued.

An intriguing area for future research is the potential role of biased agonism at these receptors in the pathophysiology of such diseases. Additionally, we reviewed the literature on A_2B_ ARs and A_3_ ARs, particularly in the context of neuroinflammation and, to a lesser extent, neurodegeneration. These receptors have been largely underexplored in these areas. However, with the development of more effective pharmacological tools, this gap is likely to be addressed in the coming years. This progress may lead to a more comprehensive understanding of the adenosine receptor function in central nervous system disorders and potentially pave the way for novel therapeutic strategies for currently unmet medical needs.

## Data Availability

Not applicable. No new data were generated.

## Abbreviations

The following abbreviations are used in this manuscript:

α-Syn	α-Synuclein
Aβ	Amyloid beta
AβPP	Amyloid-beta precursor protein
AD	Alzheimer’s disease
ALS	Amyotrophic lateral sclerosis
ALSFRS-R	Revised amyotrophic lateral sclerosis functional rating scale
APP	Amyloid precursor protein
AR	Adenosine receptor
ATP	Adenosine triphosphate
AV	Atrioventricular
BACE1	β-secretase 1
BBB	Blood brain barrier
BDNF	Brain-derived neurotrophic factor
BFCN	Basal forebrain cholinergic neurons
C9orf72	Chromosome 9 open reading frame 72
CCH	Chronic cerebral hypoperfusion
CCL	C-C motif chemokine ligand
CLEC7A	C-Type lectin domain containing 7A
CNS	Central nervous system
COPD	Chronic obstructive pulmonary disease
CREB	cAMP response element-binding protein
DAMP	Danger-associated molecular pattern
DNA	Deoxyribonucleic acid
EL	Extracellular loop
ERK	Extracellular regulated kinase
FDA	Food and drug administration
FUS	Fused in Sarcoma, also called translocated in liposarcoma protein (TLP)
GABA	Gamma-aminobutyric acid
GLT-1	Glutamate transporter 1
GPCR	G protein-coupled receptor
H_1_	Histamine 1
HD	Huntington’s disease
IFN	Interferon
IL	Interleukin
JAK	Janus kinase-
JNK	C-Jun N-terminal kinase
KO	Knock out
L-DOPA	Levo-dioxyphenylalanine
LPS	Lipopolysaccharide
LTD	Long term depression
LTP	Long term potentiation
MAPK	Mitogen activated protein kinase
MAO-B	Monoamine oxidase B
mGLU5	Metabotropic glutamate receptor 5
MPI	Myocardial perfusion imaging
MS	Multiple sclerosis
NADPH	Nicotinamide adenine dinucleotide phosphate
NECA	N-ethylcarboxamidoadenosine
NF-κB	Nuclear factor kappa B
NFT	Neurofibrillary tangle
NLRP3	NOD-, LRR- and pyrin domain-containing protein 3
NMDA	N-methyl-D-aspartate
NO	Nitric oxide
OGD	Oxygen glucose deprivation
PAMP	Pathogen-associated molecular pattern
PD	Parkinson’s disease
PLC	Phospholipase C
PKA	Protein kinase A
PKC	Protein kinase C
PRR	Pattern-recognition receptors
PS1	Presenilin-1
RNA	Ribonucleic acid; mRNA (messenger RNA)
ROS	Reactive oxygen species
SAE	Sepsis associated encephalopathy
SAP102	Synapse-associated protein 102
Shh	Sonic hedgehog
SN	*Subtantia nigra*
SOD1	Superoxide dismutase 1
STAT3	Signal transducer and transcription activator 3
SVT	Supraventricular tachycardia
TBI	Traumatic brain injury
TDP-43	Trans activation response DNA binding protein 43
TGF-β	Transforming growth factor β
TLR	Toll-like receptor
tMCAo	Transient middle cerebral artery occlusion
TNF	Tumor necrosis factor
TrKB	Tropomyosin receptor kinase B/tyrosine receptor kinase B
tPA	Tissue plasminogen activator
USA	Unites States of America
VEGF	Vascular endothelial growth factor
